# The Economic Impact of Weight Regain

**DOI:** 10.1155/2013/379564

**Published:** 2013-12-26

**Authors:** Caroline E. Sheppard, Erica L. W. Lester, Anderson W. Chuck, Daniel W. Birch, Shahzeer Karmali, Christopher J. de Gara

**Affiliations:** ^1^Centre for the Advancement of Minimally Invasive Surgery, Faculty of Medicine & Dentistry, University of Alberta, Edmonton, AB, Canada T5H 3V9; ^2^University of Alberta, Edmonton, AB, Canada T6G 2C9; ^3^University of Alberta, Institute of Health Economics, Edmonton, AB, Canada T5J 3N4; ^4^Faculty of Medicine & Dentistry, University of Alberta, Edmonton, AB, 2-590 Edmonton Clinic Health Academy, 11405-87 Avenue NW, Edmonton, AB, Canada T6G 2C9

## Abstract

*Background*. Obesity is well known for being associated with significant economic repercussions. Bariatric surgery is the only evidence-based solution to this problem as well as a cost-effective method of addressing the concern. Numerous authors have calculated the cost effectiveness and cost savings of bariatric surgery; however, to date the economic impact of weight regain as a component of overall cost has not been addressed. *Methods*. The literature search was conducted to elucidate the direct costs of obesity and primary bariatric surgery, the rate of weight recidivism and surgical revision, and any costs therein. *Results*. The quoted cost of obesity in Canada was $2.0 billion–$6.7 billion in 2013 CAD. The median percentage of bariatric procedures that fail due to weight gain or insufficient weight loss is 20% (average: 21.1% ± 10.1%, range: 5.2–39, *n* = 10). Revision of primary surgeries on average ranges from 2.5% to 18.4%, and depending on the procedure accounts for an additional cost between $14,000 and $50,000 USD per patient. *Discussion*. There was a significant deficit of the literature pertaining to the cost of revision surgery as compared with primary bariatric surgery. As such, the cycle of weight recidivism and bariatric revisions has not as of yet been introduced into any previous cost analysis of bariatric surgery.

## 1. Background

Obesity has been established as a global economic burden. Several countries have already quantified the costs associated with obesity on their healthcare systems, and unequivocally bariatric surgery has been found to be a cost-effective method for reducing obesity related costs and increasing quality of life [1–4]. However, the literature has investigated neither the cost of procedure failure rate due to weight regain or insufficient weight loss, nor the cost burden of patients returning to their original obesity status.

The rate of weight regain has been reported as ranging from 5 to 39% corresponding to a median of 20% (average: 21.1% ± 10.1%, range: 5.2–39, *n* = 10) [5–14]. Several authors have attributed this phenomenon to mechanical failure, such as pouch and stoma dilation, while others believe that the behavioural component is the main contributor to weight gain over time [8, 15, 16]. Weight recidivism can be dealt with via two facets: the patient can remain obese or an attempt at surgical revision can be undertaken. Revision can include band removal, band replacement, conversion to sleeve gastrectomy or gastric bypass, gastric bypass limb lengthening, and endoscopic techniques, each of which has an associated cost and complication risk. Many institutions in the United States will have performed multiple revisions for their patients.

The objective of this study was to identify the revision rates of bariatric procedures and only the direct healthcare costs associated with weight recidivism by performing a literature search. This study does not intend to serve as a cost analysis of the impact revision which surgery has on bariatric surgery cost-effectiveness; indirect costs to society are not included.

## 2. Methods

### 2.1. Literature Search

A Medline search was performed in June 2013 with the assistance of a health librarian at the University of Alberta using search terms: bariatric surgery, revision, recidivism, cost-analysis, and economics. For a complete list of mesh terms see the appendices.

**Figure 1:**
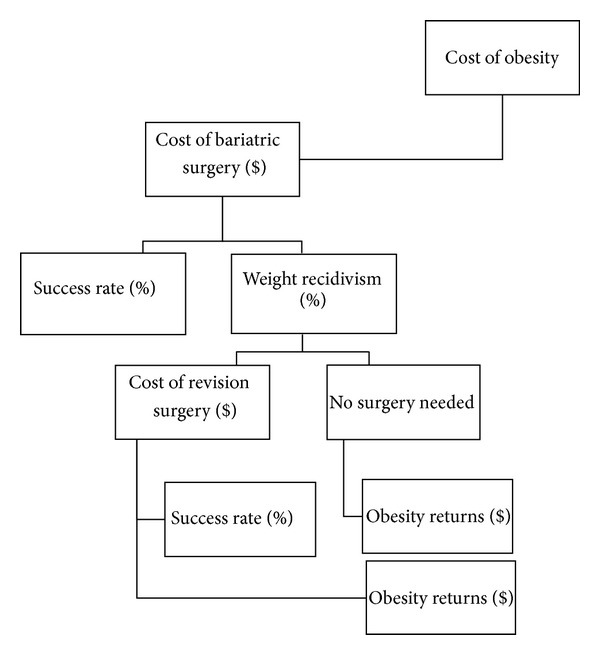
Process of primary bariatric surgery to revision surgery and their outcomes.

The preliminary search was performed to identify the cost of various bariatric surgeries, including revision procedures, and the rate of weight regain associated with these procedures. A total of 213 articles were identified. A secondary search was performed in July 2013 for articles with fail* or revision* in title along with the names of the major bariatric surgeries and limited to articles published between 2003 and 2013. This identified 198 articles. Articles were not included if they involved fundoplication, antireflux surgery, plastic surgery, revision of vertical banded gastroplasty (since it is no longer performed as a primary surgery), duplicate findings, revision of bariatric procedures due to complications such as ulcers, staple line failure, or hemorrhages, case studies, and revision of bariatric surgery due to hepatic or renal failure. The literature search was designed to investigate the data pertaining to the paradigm outlined in Figure 1.

### 2.2. Costing

Direct costs of obesity were included in this study from several countries and the literature available since 2000. Direct costs were identified using the definition described by Terranova et al., such that costs represented the costs of behavioural, pharmacological, and physician management of obesity associated comorbidities [3]. Indirect costs, such as loss of work productivity, disability payments, and loss of productive years, were not included in the calculation of obesity. The Canadian studies were converted to 2013 CAD using the Bank of Canada inflation calculator based on monthly consumer price indexes determined by Statistics Canada [17].

The cost of primary bariatric surgery was provided by Alberta Health Services Financial Department. At our institution, 61 laparoscopic adjustable gastric bands (LAGB) from 2011 to 2012, 227 laparoscopic sleeve gastrectomies (LSG), and 187 laparoscopic Roux-en-Y gastric bypasses (LRYGB) were performed from 2010 to 2012 at a mean total cost of $10,470.90, $11,934.17, and $17,882.96, respectively. These costs are a summation of average direct costs (average costs of length of stay, supplies, staff, medications, equipment, ambulance transfers, and sundries) and indirect costs (administration and support costs for each clinical service). Complication costs were estimated based on the summation of direct procedure costs and multiplied by the rate of occurrence for each bariatric procedure. Complication costs were represented as the costs per complicated patient each year. The cost of receiving any of the three mentioned bariatric procedures plus complications was calculated. Patients also attend a median of nine visits with the Weight Wise clinic multidisciplinary team before surgery. The team includes physicians, psychologists, dieticians, nurses, psychiatrists, and surgeons, whom all contribute a cost.

A basic cost of revision was based on averages provided by the operating room at our institution. The cost of the bariatric revision team was included in this calculation for a median of four visits per patient. Only a range of complication costs was provided, due to an unidentified complication rate for revision surgeries. Surgeon billing was also included in this amount. The total cost of revision surgery is based on conservative costing; not all direct costs were available. Bariatric surgeries included in this calculation are gastroplasty reversal, stomach resection, open bariatric procedures, and bariatric reversals.

### 2.3. Analysis

The bariatric procedure failure rates due to weight recidivism, total revision rate, and revision rate due to weight recidivism or insufficient weight loss were averaged and presented as average ± standard deviation (number, range), or the median was used when appropriate.

## 3. Results

### 3.1. Cost of Obesity

The quoted cost of obesity in Canada was $2.0 billion–$6.7 billion in 2013 CAD [18, 19]. Direct obesity expenditures were estimated to be 4.1% of total health expenditures in 2006 [19]. Obesity costs in Alberta alone were reported to range from $109.9–$726.4 million in 2013 CAD [20, 21]. In the United States, costs of obesity have been said to range from $98.1–209.7 billion in 2008, attributing to 20.6% of US national health expenditures [22, 23]. Other nations, such as Australia, determined that direct costs were $2788 AUD per person per annum (2005) [24].

### 3.2. Cost of Primary Bariatric Surgery

At our institution, the average cost of LAGB, LSG, or LRYGB in a year was calculated to be $13,869.95. Additionally, complications included band removal, ulceration, hemorrhage, staple line leak, anastomotic stricture, and internal hernia. The median complication rate was 1.2% (average: 2.6% ± 3.3, range: 0.3%–8.9%), equivalent to a cost of $556.83 per complicated patient each year (range: $310.33–$4,208.20). In addition to the cost of procedure and complications, a patient will incur a cost of $495.86 for visits with the bariatric team. In total approximately $14,383.32 (range: $10,984.27–$18,396.33) was spent per patient per year. To our knowledge no other Canadian literature has published the cost of initial bariatric surgery. The American literature quotes bariatric surgery to cost anywhere from $14,000 to $24,000 for all procedures [1, 2, 25].

### 3.3. Rate of Primary Revision

**Figure 2:**
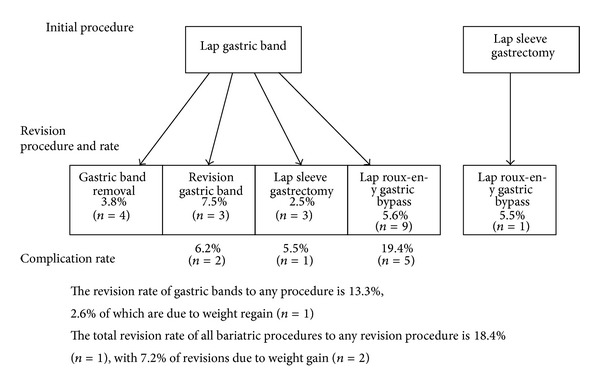
Average rate of bariatric surgery revision.

A review of the literature was performed, and 36 articles contained information on the failure rate of bariatric procedures due to weight gain/insufficient weight loss or primary and secondary revision rates (Table 1). The median percentage of bariatric procedures that fail due to weight gain or insufficient weight loss is a median of 20% (average: 21.1% ± 10.1%, range: 5.2–39, *n* = 10), and a mean of 22 ± 10 kg of regained weight between 1 and 52 months [5–14]. The rates of revision can be found in Figure 2 [5, 9, 10, 13, 14, 26–46]. The average of a second bariatric revision surgery ranges from 20.0% to 25.2%, and 13.0% to 25.0% due to weight gain [5, 13, 34, 41].

The average timeframe from primary bariatric procedure to revision was 35.4 ± 13.4 months (range: 4 days–120 months, *n* = 13) [5, 9, 10, 13, 14, 26, 27, 33, 39, 41–43, 47]. The rate of patients that regained weight but did not receive surgery or were not surgical candidates could not be identified from the literature. These patients may incur healthcare costs similar to being originally obese with comorbidities. Tucker et al. reported that 22% of revision patients required intervention due to either comorbidity recurrence or nonresolution [13]. In addition, procedures such as LAGB have immense variation in success with reducing or resolving comorbidities (28.6%–100%) such as type 2 diabetes, sleep apnea, and hypertension [48]. Failed LSG has a range of comorbidity prevalence in revision patients between 11% and 56% [42]. The costs of an obese individual with comorbidities have been identified in the Canadian literature between $600 and $1,200 per person per annum in 2013 CAD for physician costs and hospitalization [49, 50]. The American literature reports $1,700 and $2,700 in 2008 and 2005 USD, respectively [22, 23].

After revision surgery, patient's excess weight loss was 48.7%–50% after 2 years and an average of 15.2 kg lost after 1–52 months [14, 43, 51]. While not performed at our institution, LRYGB revision by limb lengthening has been reported to improve weight loss with an EWL from 26.6% to 60.9% 1 year postrevision and 68.8% after 5 years [52, 53].

Complication rates for revision surgery have been reported as either equivalent to primary bariatric surgery 29.6% versus 30.9% [31, 41, 43] or significantly higher. These complications included band slippage and erosion, hernia, infection, abdominal abscess, gastric emptying, anastomotic leak, strictures and ulcers, and weight loss failure. The average complication rate for all revision procedures ranged from 5.5% to 19.4% [5, 10, 13, 33, 35, 44, 45, 54]. More complex revision procedures such as biliopancreatic diversion have higher rates of complications of 25.3% [10]. These complication rates were also associated with longer average length of hospital stay, 2.8–3.6 days, and 8.2 days for a second revision [13, 35, 44].

### 3.4. Cost of Revision

The average surgical cost of bariatric revision at our institution is $5,624.00. Gastric band removal ranges from $895 to $1,223. Surgeon billing depending on the procedure can vary from $889.60 to $2,930.59. The cost of complications may vary from investigating ulcers and anastomotic leaks ($197.60–$228.82) to a laparotomy ($120–$650). Hospital stay is $1,483 per night or $3,178 per night in the intensive care unit (ICU). The bariatric team costs $218.18 per patient for a median of 4 visits. A total cost at our institution for bariatric revision surgery can be approximated to range from $3,485.78 to $12,617.59 CAD.

From the literature and as would be expected, a greater hospital cost was incurred by revision surgery than by primary bariatric surgery. For gastric banding a difference of $4,147 in hospital costs ($14,153 ± $14,227 versus $10,004 ± $4749, 2011 USD) and LRYGB incurs a difference of $13,257 ($35,189 versus $49,377) [35, 44]. These do not include the cost of an interdisciplinary team or costs incurred by complications.

## 4. Discussion

Thirty-six articles were found with information on bariatric revision rates. Causes of revision surgery may vary from gastric stoma dilation to lack of follow-up with the bariatric team (60%–80% of patients) [8]. The majority of these articles focused on gastric band revision, which was reported as having an increased number of reoperations, rising nearly 2-fold from 2005 to 2008 in the United States [44]. The literature pertaining to revision rates is sparse for LSG or LRYGB revision. While the cost of obesity has been thoroughly researched, there was a paucity of the literature on the cost of these reoperations. Furthermore the costs elicited were from American sources, limiting the ability to extrapolate Canadian costs for revision.

Regardless of nationality, obesity comprises a substantial amount of the countries' healthcare expenditures. In addition, Peeters et al. reported that life expectancy of obese men and women is decreased by 5.8 and 7.1 years, respectively [55]. A costly component is bariatric surgery yet it is considered the most successful treatment for this disease [56]. In Canada alone, estimated 1100–1200 bariatric surgeries are performed every year [57]. The cost of bariatric surgery has been well established as a long term cost-effective method for treating obesity due to the reduction in comorbidity management costs, despite a front loaded cost of $14,000–$24,000. The cost estimates calculated at our institution and within the literature are similar.

An 18%, 58%, and 82% reduction in obesity related costs could be observed at 12 months, 13–24 months, and 25–36 months after bariatric surgery, respectively [58]. Monk et al. reported cost savings of $182.10 a month in pharmaceutical use 6 months postop ($317.30 preop versus $135.20 postop, in 2004 USD) [59]. Cost-effectiveness is measured by determining the incremental cost-effectiveness ratio, which contrasts incremental costs with incremental health benefits (increased years of life). A lower incremental cost-effectiveness ratio indicates that the same unit of outcome can be achieved at a lower cost [20]. The Canadian Agency for Drugs and Technologies in Heath (*CADTH*) determined that all primary bariatric procedures corresponded with an incremental cost-utility ratio ranging from $6,500 to $12,000 per additional quality-adjusted life years (QALY), compared to nonsurgical treatment over a life span [57]. These calculations included revision surgery as part of the complications of surgery occurring within one year postop, not weight recidivism, and were based on merging the cost-effectiveness of bariatric surgery found in several articles. The literature from the United States is similar to an incremental cost-effectiveness ratio of $6,600 per QALY for LRYGB and $6,200 for LAGB [1]. Cost savings of $900 USD per month can be seen as early as 13 months postop from laparoscopic bariatric surgery (in 2008) [2]. These cost savings account for a reduction in two-thirds of medical expenses associated with obesity [60].

Revision surgery has not been taken into account when calculating these cost savings. The rate of weight recidivism uncovered in the literature is approximately equivalent to our institution's estimated rate of 10–20%. There are several types of revision procedures that are performed when weight loss is unsuccessful. Revision of a primary surgery on average ranges from 2.5% to 18.4%, and depending on the procedure accounts for an additional cost of $14,000–$50,000. It is projected that our institution would incur a similar cost due to revision. This accounts for a substantial cost to the system and does not include the cost of complications, the cost of subsequent revisions (20.0%–25.2%), or continuing in a bariatric intervention program. The rate of complications has been predominantly reported as being significantly higher than primary bariatric surgery (5.5%–19.4%) and would indicate that the cost of complications would also be greater. While complication rates vary between procedures, according to the *CADTH*, AGB has been recognized as requiring procedure reversals or conversions more often than RYGB, regardless of the decreased risk of anastomotic ulceration, stricture, or hernia [57]. However, the time from the primary surgery to revision plays a role in decreasing costs if the patient was successful in losing weight due to the primary surgery. A discount rate applies to the expected rate of revision, to allow for a decrease in costs by 5% each year of successful weight loss [57]. This would correspond to a 5% reduction in costs for the average of 2.9 ± 1.1 years (35.4 ± 13.4 months) until revision is required.

While not performed at our institution, there are several endoscopic revision treatments, such as sclerotherapy, Bard EndoCinch Suturing system, Incisionless Operating Platform, StomaphyX, OverStitch, OTSC-Clip, and sodium morrhuate, and more complex procedures, such as the biliopancreatic diversion with or without duodenal switch (BPD-DS) that incur another cost on the healthcare system [15, 61]. In particular, BPD-DS has been noted to have a complication rate of 25% as a revision procedure and requires an expert skill set to perform.

Given that the literature of the revision rates is not Canadian, it would not be accurate to apply Canadian costing to revision rates that would be expected to be for a substantially larger influx of bariatric patients per year at an American institution. Our institution is budgeted for approximately 250 bariatric procedures a year and an additional 35 of these spots corresponding to revision patients from our institution and others. Due to these barriers, we are not in a position to present accurate cost analysis of Canadian bariatric surgery.

Revision surgery is successful in achieving weight loss even following initial weight recidivism [62], which may factor in overall cost savings. However, no literature exists on the cost of patients who return to their initial weight with comorbidities and are either not willing to have another operation or are not revision candidates. How this weight recidivism plays into the economic analysis of bariatric surgery and societal obesity costs has not yet been investigated to our knowledge.

All of the branches of Figure 1 encompass costs, not simply to the healthcare system but to society as a whole. Despite the amount of funding and research available for bariatric surgery, a paucity exists in the bariatric revision literature. Reoperations represent a substantial cost to the healthcare system and should be incorporated into cost-benefit analysis for bariatric surgery to obtain accurate data for this treatment.

## 5. Conclusion

In summary, when making a commitment to offer bariatric surgery services, costs need to account for an inevitable proportion of patients who will fail in primary surgery and will need a multitude of additional strategies to deal with their weight recidivism/regain. These range from simple diet/lifestyle interventions through to endoscopic manoeuvers and significantly major revisional surgery. Consequently, a significant dearth of information exists regarding these inevitable costs. Directions will be taken to calculate the revision rate at our institution and the associated costs.

## Figures and Tables

**Table 1:** The literature search for rates of revision by bariatric procedure.

Author, location	Surgery	Weight recidivism rate (%)	Timeframe to weight recidivism (months)	Revision surgery	Revision rate (%)	Revision rate (weight recidivism) (%)	Timeframe to revision (months)	Complication rate (%)	2nd revision surgery	Timeframe to 2nd revision (months)	2nd revision rate (%)
Abu-Gazala and Keidar [9], Israel	LAGB	22.2	—	LSG, LRYGB, LBPD-DS, and LBPD	Total: 6.6, LSG: 2.64, LRYGB: 2.64, and LBPD-DS: 1.32	—	36	—	—	—	—
Acholonu et al. [26], United States	LAGB	—	—	LSG	2.7	—	34.7 (16–60)	—	—	—	—
Ardestani et al. [5], United States	LAGB	24.4	24	LAGB revision (reband or reposition)/LAGB Removal	15.3/2.6	84.2 of LAGB revisions	Revision in 21.5 (0–49), conversion in 27.6 (11–48)	Revision 4.3, conversion 10.5	LAGB revision/reLAGB-RYGB	—	LAGB revision: 22.6, reLAGB-RYGB: 5.3
Bardsley and Hopkins [27], Australia	LAGB	—	—	All procedures	13	2.6	3–36	—	—	—	—
Biertho et al. [10], United States	LAGB	17.4	—	ReLAGB (with BPD), LAGB removal (with LRYGB)	Total: 4.7, reLAGB: 2, LAGB removal: 0.2, reLAGB-BPD: 0.5, LAGB removal-LRYGB: 1.9	—	22.8 ± 2.4	ReLAGB: 8.1, LAGB removal-LRYGB: 6.3, ReLAGB-BPD: 25.3	—	—	—
Brolin and Asad 2009 [28], United States	All procedures	—	—	All procedures	9.8	6.6	—	33	—	—	—
Brolin and Cody 2007 [53], United States	LRYGB	—	—	Distal LRYGB	—	—	—	18.5	—	—	—
Christou et al. [6], Canada	LRYGB	18/35	60/120	—	—	—	—	—	—	—	—
Deylgat et al. [29], Belgium	LAGB/LSG	—	—	LRYGB	—	LAGB: 38.9, LSG: 8.3	—	—	—	—	—
Hedberg et al. [54], Sweden	LAGB	—	—	RYGB	—	—	—	8	—	—	—
Hii et al. [31], Australia	All procedures/LAGB	—	—	All procedures/RYGB	All procedures: 18.4, RYGB: 0.7	—	—	Equivalent rate to primary surgery	—	—	—
Jennings et al. [32], UK	LAGB	—	—	LRYGB	14.6	—	—	—	—	—	—
Langer et al. [14], Austria	LSG	—	—	LRYGB	—	6.8	33 (15–70)	—	—	—	—
Magro et al. [8], Brazil	LAGB	18.8	24	—	—	—	—	—	—	—	—
Mognol et al. [33], France	LAGB	—	—	LRYGB	—	9.4	42 (7–74)	14.3	—	—	—
M$\ddot{\text{u}}$ller et al. [34], Switzerland	LAGB	—	—	LRYGB, LAGB	LRYGB: 40, reLAGB: 60 of revised LAGB	—	—	—	ReLAGB, LAGB removal-LRYGB, and distal LRYGB	—	ReLAGB: 45 (25 for weight), LAGB removal-LRYGB: 2.2, and distal LRYGB: 20 (13 for weight)
Nguyen et al. [35], United States	LAGB	—	—	ReLAGB, LAGB removal	ReLAGB: 0.76, LAGB removal: 0.87	—	—	18.8	—	—	—
Patel et al. [36], United States	All procedures (except LAGB)	—	—	All procedures	2.5	—	—	—	—	—	—
Poyck et al. [37], Netherlands	LAGB	—	—	BPD-DS (LRYGB)	—	17	—	—	—	—	—
Roller and Provost [38], United States	LAGB	—	—	LRYGB	—	59.5 of LAGB revisions	—	—	2nd revision: 29.6, multiple revisions: 41.7	—	—
Shimizu et al. [7], United States	RYGB	10–20	—	—	—	—	—	—	—	—	—
Snyder et al. [11], United States	LAGB/LRYGB	34/5.2	—	—	—	—	—	—	—	—	—
Spivak et al. [39], United States	LAGB	—	—	LRYGB	—	1.7	28.2 (11–46)	—	—	—	—
Suter et al. [40], Switzerland	LAGB	—	—	All procedures	30.1	—	—	—	—	—	—
te Riele et al. [41], Netherlands	LAGB	—	—	LRYGB	14.1	11.4	66.2 (12.7–120)	Equivalent rate to primary (29.6 versus 30.9)	revision due to complications, none for weight gain	—	20
Tucker et al. [13], United States	LAGB	39	—	—	Total: 16.5, LAGB removal: 9.1, LAGB removal-LSG: 3.6, and LAGB removal-LRYGB: 2.3	7.8	21.8 ± 13.9 (4 days–47 months)	15.2	2nd revision/3rd revision/4th and 5th	13.2 (3 days–44.7 months)/21.2 (12–28.5)	20/7.5/2.5
Van Dessel et al. [47], Belgium	LSG/LAGB	—	—	LRYGB	—	100/50 of revisions	54	—	—	—	—
van Rutte et al. [42], Netherlands	LSG	—	—	LRYGB	5.5	—	18 (10–21)		—	—	—
Van Wageningen et al. [43], Netherlands	LAGB	—	—	LRYGB	7.6	62	54 ± 22.8	Equivalent rate to primary	—	—	—
Worni et al. [44], United States	LAGB	—	—	LRYGB	0.6	—	—	3.7	—	—	—
Yazbek et al. [45], Canada	LAGB	—	—	LSG	—	57.7 of LAGB revisions	—	5.5	—	—	—
Yimcharoen et al. [12], United States	RYGB	10–20	60–120	—	—	—	—	—	—	—	—
Zagzag et al. [46], United States	LAGB	—	—	All procedures	1.76	—	—	—	—	—	—

## Appendices

### A. Preliminary Search June 28, 2013

1. exp Bariatric Surgery/
2. (bariatric surgery or LAGB or gastric band* or lap band* or lap-band* or lapband* or roux-en-y or vertical sleeve or sleeve gastrectom* or gastric bypass* or jejuno-ileal bypass* or Jejunoilea* bypass*).mp.
3. Obesity, Morbid/su [Surgery]
4. 1 or 2 or 3
5. Reoperation/
6. revision.tw.
7. (regain* or recidivism).tw.
8. ((weight or pounds or lbs or kgs or kilograms or percent*) adj6 (gain or gained)).tw.
9. Weight Gain/
10. 5 or 6 or 7 or 8 or 9
11. 4 and 10
12. exp “Costs and Cost Analysis”/
13. (cost* or economic* or expenditures or price or fiscal or financial or burden or efficiency or pay or valuation or pharmacoeconomic or spending).ti.
14. (economic adj1 (evaluat* or analys* or study or studies or assess* or consequence*)).tw.
15. (cost-benefit or benefit-cost or cost effectiv* or cost utility).tw.
16. (cost minimization or cost minimisation or cost consequence* or cost offset*).tw.
17. ((cost or costs) adj2 analys*).tw.
18. “cost of illness”.tw.
19. or/12–18
20. 11 and 19
21. (surgery or bypass or sleeve or band* or lap-band or plicaton or roux-en-y).ti.
22. (gain or gained or regain* or recidivism or revision* or fail* or reoperation).ti.
23. pregnan*.ti.
24. (11 and 21 and 22) not 23
25. (213 articles).

### B. Secondary Search July 15, 2013

1. exp Bariatric Surgery/
2. (bariatric surgery or LAGB or gastric band* or lap band* or lap-band* or lapband* or roux-en-y or vertical sleeve or sleeve gastrectom* or gastric bypass* or jejuno-ileal bypass* or Jejunoilea* bypass*).mp.
3. Obesity, Morbid/su [Surgery]
4. 1 or 2 or 3
5. 4 and (revision or (fail* not (heart fail* or liver fail* or respiratory fail* or renal fail* or hepatic fail* or kidney fail*))).ti.
6. limit 5 to yr = “2003–2014”
7. (198 articles).
